# A global systematic overview of socioeconomic factors associated with antidiabetic medication adherence in individuals with type 2 diabetes

**DOI:** 10.1186/s41043-023-00459-2

**Published:** 2023-11-07

**Authors:** Christian Ming Studer, Marie Linder, Laura Pazzagli

**Affiliations:** 1 Department of Chemistry and Applied Biosciences, Institute for Pharmaceutical Sciences, ETH Zurich, Zurich, Switzerland; 2 Centre for Pharmacoepidemiology, Department of Medicine Solna, Karolinska Institutet, Stockholm, Sweden; 3 Clinical Epidemiology Division, Department of Medicine Solna, Karolinska Institutet, Stockholm, Sweden

**Keywords:** Diabetes mellitus, Type 2, Socioeconomic factors, Medication adherence, Systematic review

## Abstract

**Background:**

Antidiabetic medication adherence is a key aspect for successful control of type 2 diabetes mellitus (T2DM). This systematic review aims to provide an overview of the associations between socioeconomic factors and antidiabetic medication adherence in individuals with T2DM.

**Methods:**

A study protocol was established using the PRISMA checklist. A primary literature search was conducted during March 2022, searching PubMed, Embase, Web of Science, as well as WorldCat and the Bielefeld Academic Search Engine. Studies were included if published between 1990 and 2022 and included individuals with T2DM. During primary screening, one reviewer screened titles and abstracts for eligibility, while in the secondary screening, two reviewers worked independently to extract the relevant data from the full-text articles.

**Results:**

A total of 15,128 studies were found in the primary search, and 102 were finally included in the review. Most studies found were cross-sectional (72) and many investigated multiple socioeconomic factors. Four subcategories of socioeconomic factors were identified: economic (70), social (74), ethnical/racial (19) and geographical (18). The majority of studies found an association with antidiabetic medication adherence for two specific factors, namely individuals’ insurance status (10) and ethnicity or race (18). Other important factors were income and education.

**Conclusions:**

A large heterogeneity between studies was observed, with many studies relying on subjective data from interviewed individuals with a potential for recall bias. Several socioeconomic groups influencing medication adherence were identified, suggesting potential areas of intervention for the improvement of diabetes treatment adherence and individuals’ long-term well-being.

**Supplementary Information:**

The online version contains supplementary material available at 10.1186/s41043-023-00459-2.

## Background

According to the International Diabetes Federation, the diabetes mellitus epidemic affected about 537 million adults aged 20–79 years worldwide in 2021 and about 90% of all diabetes cases are considered to be type 2 diabetes mellitus (T2DM) cases [1]. The number is predicted to rise to 643 million by 2030 and 783 million by 2045, with the largest proportion based in low- to middle-income countries [1]. Among the causes of this rapidly growing epidemic, there are population ageing, economic advances, rise of obesity and sedentary lifestyle, as well as excessive consumption of sugar and fat [2, 3].

Adequate treatment of the disease is of great importance, as uncontrolled T2DM is strongly associated with cardiovascular risk factors, the number one cause of death for individuals with T2DM, contributing to the dysmetabolic syndrome [4]. Uncontrolled T2DM can also lead to diabetic kidney disease [5], diabetic retinopathy, neuropathy and an increased risk for periodontitis [1].

Therapeutic approaches are highly individualized and based on the severity of the disease, the estimated life expectancy and comorbidities [6–9]. An essential indicator of T2DM severity and efficacy of treatment is the glycated haemoglobin (HbA1c) value [10]. Initial recommendations for reaching and maintaining a target HbA1c value include lifestyle modifications such as reducing sedentary lifestyle, increasing physical activity, smoking cessation, reduction in the alcohol consumption and, most importantly, reduction in the obesity and diet adjustments. If these changes do not suffice, medication treatment is recommended, starting with medications without risk of hypoglycaemia, followed by medication with risk of hypoglycaemia, up to administration of insulin [6–9].

Adherence to recommended medications plays a key role for a successful treatment strategy in terms of efficacy. Poor medication adherence is encountered frequently and is highly associated with increased morbidity, mortality, higher costs and more hospitalizations [11–17], and it may be affected by socioeconomic and demographic factors influencing individuals’ lives. A review looking at the association between ethnicities and migration background and non-adherence was conducted in 2009, but led to inconclusive results as the number of included studies was too small and differences in study conduction were substantial [18]. More recent studies focused on developing nations [19], low- to middle-income countries [20] or Asian countries [17]. A positive association between education and adherence was found in some studies [17, 20], while others found an association with non-adherence for both employed and individuals with low income [20]. In contrast, another review found that employment is not associated with a difference in adherence [17].

The inconsistences of earlier findings and the lack of more comprehensive reviews call for a systematic review aiming to provide a global perspective of the relationships between antidiabetic medication adherence and socioeconomic factors in individuals affected by T2DM. Specifically, socioeconomic factors of interest are all social determinants of health as defined by the World Health Organization which include all non-medical factors influencing health outcomes such as medication adherence. The current review proposes an up-to-date overview of the research conducted on the topic and related findings, and provides a classification of the results and the direction of the findings based on two major groups of socioeconomic factors (economic/social and ethnical/geographical). Finally, it aims to support practitioners to identify vulnerable populations of patients and needs for interventions and further research.

## Materials and methods

The study aimed to retrieve all available evidence on the associations between socioeconomic factors and antidiabetic medication adherence in individuals with T2DM. Factors included in the literature review may have heterogeneous naming, definitions and categorizations, and the reporting of the results was based on an overall classification of socioeconomic factors, which divides them into economic/social or ethnical/geographical.

Additionally, medication use in published articles was referred to using terms such as ‘adherence’, ‘compliance’, ‘concordance’ and ‘persistence’. Some of these terms are used inconsistently or often interchangeably and are also dependent on the study design [14, 21, 22]. In this systematic review, adherence was used as an umbrella term according to Vries et al. (2012) and defined as a combination of a patient’s medication initiation, implementation, persistence and discontinuation [14].

A study protocol was established following the PRISMA checklist. A literature search was conducted using the major online databases containing biomedical and life sciences literature, namely PubMed, Embase, Web of Science, as well as on the grey/unpublished literature databases WorldCat and the Bielefeld Academic Search Engine (BASE). Free-text terms used for the search consisted of three main blocks: (diabetes mellitus type 2) AND (socioeconomic factors) AND (medication adherence). A full list of terms can be found in Supplementary Material, Appendix 1. Multiple synonyms and variations were used to supplement the main keywords to increase the probability of retrieving relevant articles. Medical Subject Heading (MeSH) terms and Emtree were utilized for labelled articles search. The systematic review included original articles investigating the association between socioeconomic factors and antidiabetic medication use in individuals with T2DM published in English between 1 January 1990 and the extraction date, 15 March 2022. Articles excluded were case studies/series, as well as literature reviews, and articles that did not clearly state whether the population was including individuals with T2DM.

All articles identified from the major online databases and the grey literature were extracted into a single EndNote library. EndNote was used to automatically remove duplicates while the remaining articles were manually screened. Titles and abstracts of the articles were primarily screened for eligibility by a first reviewer. In case of uncertainties, a second reviewer examined the articles to verify eligibility for the secondary screening. Additional articles were manually selected by the first reviewer, screening original articles and literature reviews found during the primary screening. Finally, the articles included in the secondary screening were searched for additional references.

The secondary screening was conducted in parallel and independently by the two reviewers. The full text was evaluated, and prespecified information was extracted including study design, type of data collection, definition of adherence used, type of medication (insulin/oral antidiabetic), socioeconomic factors influencing adherence, the direction of the association, study period, geographical setting, number of participants, special subpopulations studied and a quality assessment. The National Heart, Lung and Blood Institute of Health (NHLBI) quality assessment tool [23] was used since it is suitable for the evaluation of both experimental and observational studies. In case of inconsistencies in the data extraction, the two reviewers elaborated their points, and when no common ground could be found, a third reviewer was consulted to reach a final assessment.

The results from the systematic review were categorized into economic/social and ethnical/geographical factors, and within these two groups, factors were classified into additional main domains and subgroups. Finally, the included studies were grouped based on the direction of the reported associations between socioeconomic factors and antidiabetic medication adherence (increase, no association, decrease), and study design.

## Results

**Fig. 1:**
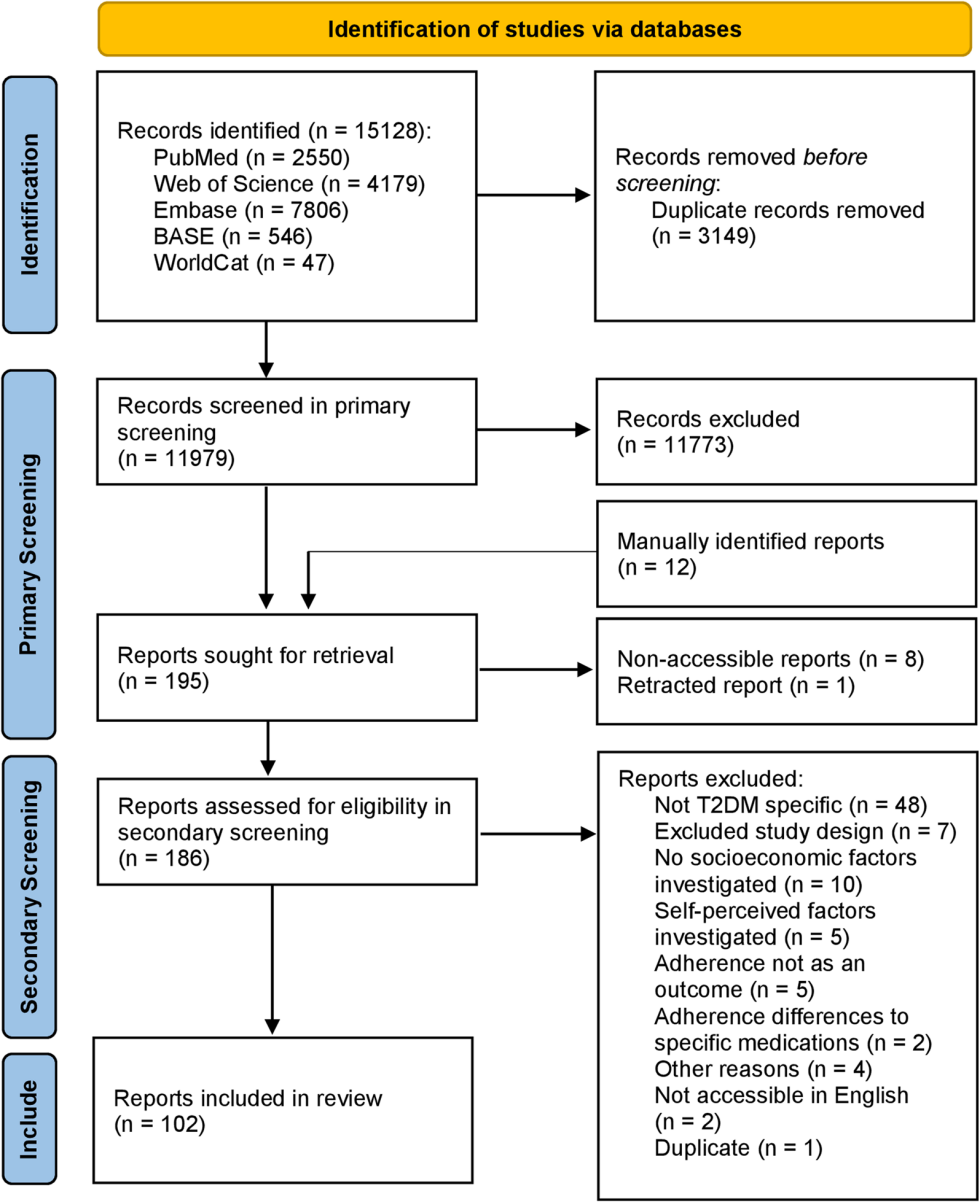
PRISMA flowchart of the included studies [120]. Adapted from the 2020 PRISMA statement

The primary screening, in which only the title and the abstract of the articles were considered, was conducted by the first reviewer, and the complete PRISMA flowchart of the studies included/excluded is shown in Fig. 1. A total of 15,128 articles were retrieved from searching the different online databases. After automated removal of duplicates, 11,979 articles were left for primary manual screening, and after that, 186 articles remained eligible for the secondary screening conducted independently by the two reviewers.

In the secondary full-text screening, 102 articles were found to be eligible. The reasons for exclusion were that the articles were not T2DM-specific (*n* = 48), the studies were conducted using an excluded study design (*n* = 7), no socioeconomic factors were investigated (*n* = 10), only self-perceived factors were investigated (*n* = 5), adherence was not an outcome (*n* = 5), adherence differences between antidiabetic medication types were investigated (*n* = 2), or the studies were not on topic for other reasons (*n* = 4). Additionally, during the full-text screening 8 articles were not accessible, 1 article was retracted, 2 articles were not available in English, and 1 article was a duplicate.

In total, 73 of the articles included had a cross-sectional study design, 23 were cohort studies, 5 were experimental and 1 was a case–control study. Categorized by continent where the study was conducted, a total of 15 studies were in Africa, 42 in Asia, 4 in South America, 1 in Central America, 29 in North America, 5 in Europe and 6 in Oceania. In total, 67 studies were identified with fewer than 500 participants, 18 studies with more than 500 participants but less than 5000 and 17 studies with more than 5000 participants. A total of 72 studies looked at all types of medication used in T2DM, 26 studies investigated individuals using oral antidiabetic medication and 4 studies investigated individuals using insulin. Utilizing the NHLBI quality assessment tool [23] led to 25 studies rated as good, 53 studies rated as fair and 24 studies rated as poor.

### Economic and social factors

A total of 70 studies investigated associations between medication adherence and one or more economic factors, while a total of 74 studies investigated social factors. The complete results with all references are given in Table 1 where the studies are reported by type of socioeconomic factor investigated, study design and direction of the association assessed (increase, no association, decrease).

**Table 1:** Economic and social factors investigated in the studies included in the systematic review

Economic and social factor	Subgroups	Direction of the association with antidiabetic medication adherence		
		Increase	Equal	Decrease
**Socioeconomic status**	Socioeconomic status	CS: **4** [24–27]	CS: **7** [28–34] C: **1** [35]	
**Occupational status**	Occupational status		CS: **16** [25, 32, 36–49] C: **1** [50] I: **1** [51]	
	Employed			CS: **1** [52] C: **1** [53]
	Unemployed	CS: **1** [54]	CS: **1** [55]	CS: **1** [56]
	Retired			CS: **1** [57]
	Housewife	CS: **1** [33]		
	Type of employment		CS: **1** [58]	
	Agricultural job			CS: **1** [31]
**Income**	High income	CS: **7** [42, 52, 59–63] C: **1** [64] RCT: **1** [65]	CS: **14** [25, 37, 41, 44, 46, 54, 58, 66–72] I: **1** [51]	CS: **1** [73]
	Daily paid worker			CS: **1** [31]
	Financial hardship			CS: **6** [74–79]
	High medication cost*		CS: **1** [80]	CS: **2** [67, 81]
**Housing type**	Housing status			CS: **1** [82]
	Homeless			C: **1** [83]
	Owning a house		CS: **1** [45]	
**Insurance Status**	Insurance status		CS: **4** [71, 72, 84, 85]	
	Medicaid			CS: **1** [77] C: **3** [86–88]
	Medicare			C: **2** [86, 88]
	None			CS: **3** [45, 67, 76] C: **1** [87] RCT: **1** [65] I: **1** [89]
	Capitated health plan			CC: **1** [90]
	Commercial insurance	CS: **3** [42, 58, 91]		
	Having health coverage	I: **1** [51]		
	Higher co-payment			C: **4** [92–95] C:**1** [50] RCT: **1** [65]
**Economic support**	Social security			CS: **1** [71]
	Economic support by relatives			CS: **1** [76]
**Transportation availability**	Transportation availability		CS: **1** [36]	
**Civil status**	Civil status		CS: **20** [25, 29, 31, 36, 37, 39, 42, 45, 48, 50, 54, 62, 66, 75, 82, 96–100] C: **1** [83]	
	Married	CS: **4** [27, 56, 101, 102] C: **1** [53]		CS: **1** [70]
	Divorced			CS: **1** [103]
	Widow			CS: **1** [103]
	Single			CS: **2** [71, 104]
**Living arrangement**	Living status		CS: **4** [30, 36, 52, 105] RCT: **1** [106]	
	Smaller family size	CS: **1** [91]		
	Living alone	CS: **1** [45]		
**Education**	Education status		CS: **36** [25, 28, 30, 31, 37, 43–47, 49, 52, 54, 56, 59–61, 67, 70–73, 75, 81, 82, 85, 97, 98, 101, 107–113] C: **2** [50, 114] I: **1** [51] RCT: **1** [106]	
	Higher education	CS: **14** [24, 27, 36, 38, 41, 42, 55, 63, 91, 100, 102, 115–117] C: **2** [53, 64] RCT: **1** [26]		CS: **1** [74]
	Higher education in immigrants	CS: **1** [85]		
	Illiterate*		CS: **5** [32–34, 46, 118]	CS: **3** [48, 62, 100]
	Numeracy	CS: **2** [72, 118]		
	Health literacy	CS: **2** [59, 72] C: **1** [119]	CS: **1** [60]	
	Disease knowledge	CS: **1** [62]	CS: **2** [71, 115]	
**Caste**	Caste		CS: **1** [46]	
**Religion**	Religious status	CS: **1** [33]	CS: **5** [32, 36, 37, 48, 81]	
	Hindu			CS: **1** [46]
Family support (social)	Family support status	CS: **1** [27]	CS: **1** [115]	

Factors marked with *—a study has found an association, but did not state in which direction [105]

Type of study—CS: Cross-sectional, C: Cohort, I: Interventional, RCT: Randomized controlled trial

Bolded numbers—Number of articles

Number in brackets—References to the articles

#### Socioeconomic status and occupational status

Three studies conducted in Asia [24–26] and 1 conducted in Egypt [27] concluded that the socioeconomic status of an individual is positively associated with medication adherence. In contrast, 7 Asian studies [28–34] and 1 study from New Zealand [35] did not find any difference in adherence. Two studies conducted in Japan [52] and Germany [53] concluded that employed individuals were less likely to be adherent than non-employed individuals. Three studies from Libya [54], Ghana [55] and Nigeria [56] investigating the association between unemployment and adherence showed results in different directions. Retirement was associated with decreased adherence in a Malaysian [57] study, while stay-at-home housewives were more likely to be adherent to their medication, according to an Indian study [58]. A total of 18 studies did not find any association between occupation and medication adherence [25, 32, 36–51].

#### Income and housing type

Five Asian studies [42, 52, 61–63] as well as 4 studies from the USA [59, 60, 64, 65] found a positive association between income and medication adherence. Opposing this trend, a single Malaysian study [73] found a negative association between higher income and medication adherence, while 15 studies across the globe found no association in either direction [25, 37, 41, 44, 46, 51, 54, 58, 66–72]. An Indian study found a negative association between individuals being paid on a daily basis and their adherence to medication [31]. Six studies [74–79] found that individuals going through financial hardship were less likely to adhere to their medication regimen. Two studies conducted in Africa [67, 81] also found a negative association between higher medication cost and adherence, whereas a Papua New Guinean study found no association [80]. A US study found that homeless individuals were less likely to be adherent [83], while an Iranian study did not find any difference in adherence between homeowners and non-homeowners [45]. ‘Housing status’ was found to be associated with decreased adherence in a Sudanese study [82]; however, the study did not clearly define the term ‘housing status’.

#### Insurance status, economic support and transportation availability

Ten US studies found that individuals were less likely to be adherent when they had no insurance [65, 87, 89], had a capitated health plan [90], or were registered with Medicaid [77, 86–88] and Medicare [86, 88]. Also, higher co-payments were associated with less adherence in 5 US studies and 1 study conducted in Bosnia and Herzegovina [50, 65, 92–95]. Likewise, commercial insurance [58] and having health coverage [51] were associated with increased adherence in 2 US studies. In other countries, a similar trend was observed, i.e. having no insurance was associated with lower adherence in 3 studies conducted in Iran [45], Nigeria [67] and Mexico [76], while commercially insured individuals in Israel [91] and in the United Arab Emirates [42] were more likely to adhere to their medication plan. Individuals with T2DM receiving social security were less likely to be adherent according to 1 US study [71], as well as individuals relying on economic support provided by relatives in 1 Mexican study [76]. Transportation availability was not associated with a change in adherence in 1 study conducted in the United Arab Emirates [36].

#### Civil status and living arrangement

Five studies from different countries concluded that married individuals are more likely to be adherent to their medication [27, 53, 56, 101, 102]. Matching these results, 1 Iranian study reported lower adherence in divorced and widowed individuals [103] and 2 US studies reported less adherence for single individuals [71, 104]. One Brazilian study opposes these findings, reporting an association between married individuals and decreased adherence [70]. However, a total of 21 studies did not find any association between civil status and medication adherence [25, 29, 31, 36, 37, 39, 42, 45, 48, 50, 54, 62, 66, 75, 82, 83, 96–100], the majority conducted in Asia [25, 29, 31, 36, 42, 45, 54, 62, 75, 98–100] and Africa [37, 48, 82, 97, 115].

One Israeli study found that smaller family size was associated with higher medication adherence [91], 1 Iranian study concluded that individuals living alone were more likely to adhere to their medication regimen [45], and 4 Asian [30, 36, 52, 106] and 1 African study [105] did not find any association between living arrangement and medication adherence.

#### Education

Higher education was associated with higher adherence in 17 studies [24, 26, 27, 36, 38, 41, 42, 53, 55, 63, 64, 91, 100, 102, 115–117], of which 11 were conducted in Asia [24, 26, 36, 41, 42, 63, 91, 100, 102, 116, 117], 4 in Africa [27, 38, 55, 115], 1 in Europe [53] and 1 in the USA [64]. One study conducted in the USA found that higher education only had a positive association with adherence when the patient had an immigrant background [85]. Three studies from Iran [100], India [62] and Ghana [48] concluded that illiterate individuals were less likely to be adherent. One Cameroonian study found a negative association between higher education and adherence [74]. The majority [39] of the studies including education did not find any association between this factor and adherence [25, 28, 30, 31, 37, 43–47, 49–52, 54, 56, 59–61, 67, 70–73, 75, 81, 82, 85, 97, 98, 101, 106–114]. Twenty-two of these studies were conducted in Asia [25, 28, 30, 31, 43–47, 49, 52, 54, 61, 73, 75, 98, 101, 106, 107, 111, 113, 114], 6 in Africa [37, 56, 67, 81, 82, 97], 6 in the USA [51, 59, 60, 71, 72, 85], 3 in Europe [50, 110, 112], 2 in South America [70, 108] and 1 in Australia [109]. Two US studies found an association between numeracy skills and higher adherence [72, 118]. Health literacy was also associated with better adherence in 2 US studies [59, 72] and 1 Japanese study [119], while a third US study did not find any association [60]. One Indian study found that better disease knowledge was associated with better adherence [62], though one Nigerian [115], as well as one US study [71] did not find any association between these two factors.

#### Caste and religion

One Indian study concluded that there was no association between caste and medication adherence [46] while another Indian study found a positive association between being religious and adherence [33]. Opposing these findings, another Indian study found that Hindu beliefs were associated with reduced adherence [46], but 3 African studies [37, 48, 81], as well as 2 Asian studies [32, 36] did not find any association between religion and adherence.

#### Family support (social)

Individuals who received social support by their families were more likely to be adherent, according to 1 Egyptian study [27], while another study conducted in Ghana [115] did not find any association.

### Ethnical and geographical factors

All studies looking at ethnical and geographical factors are given in Table 2 where the studies are reported following the same criteria used for Table 1.

**Table 2:** Ethnical and geographical factors investigated in the systematic review

Ethnical and geographical factors	Subgroups	Direction of the association with antidiabetic medication adherence		
		Increase	Equal	Decrease
**Ethnicity/race**	Ethnicity	CS: **1** [121]	CS: **5** [39, 43, 72, 107, 122] RCT: **2** [123, 124]	
	Non-white			CS: **1** [60] C: **2** [104, 125]
	Non-European			C: **1** [126]
	African-American			C: **1** [127]
	Black			C: **4** [83, 86, 88, 128] CC:**1** [90] I: **2** [51, 89]
	Asian	CS: **1** [36]		
	Malay			CS: **1** [111]
	Indian	CS: **1** [111]		
	Chinese		C: **1** [124]	CS: **1** [99]
	Japanese		C: **1** [124]	
	Filipino			C: **1** [124]
	Saudi Arabian	CS: **1** [101]		
	Arab non-Emirati	CS: **1** [36]		
	Pacific Islander			C: **3** [35, 122, 124]
	Maori			C: **3** [35, 37, 122]
	Latin-American/Hispanic			CS: **1** [121] C: **2** [88, 129]
	Native Hawaiian			CS: **1** [124]
**Country of birth**	Foreign-born		CS: **1** [85]	
	US-born			CS: **1** [71]
**Acculturation**	Acculturation	CS: **1** [39]		
	Believe in traditional Chinese medicine			CS: **1** [39]
**Accessibility to** **health care**	Accessibility to health care*			
	Distance to healthcare provider			CS: **3** [31, 61, 101]
**Area of residence**	Area of residence		CS: **5** [31, 80, 98, 100, 130] C: **1** [122]	
	Rural	C: **1** [104]		
	Urban			CS: **2** [45, 58]
	IMD quintile	C: **1** [125]		
	Neighbourhood deprivation			CS: **2** [37, 77]
	Socioeconomic living area		C: **1** [126]	
**Regional differences**	Geographical area	C: **1** [104]	CS: **2** [41, 110]	
	Southern United States			C: **1** [95]

Factors marked with *—a study has found an association, but did not state in which direction [105]

Type of study—*CS* cross-sectional, *C* Cohort, *I* interventional, *RCT* randomized controlled trial

Bolded numbers—Number of articles

Number in brackets—References to the articles

*IMD* indices of multiple deprivation

#### Ethnicity/race

A total of 19 studies looked at ethnical and racial differences in antidiabetic medication adherence [35–37, 39, 43, 51, 60, 71, 72, 83, 85, 86, 88–90, 99, 101, 104, 107, 111, 121–128, 131]. Fifteen studies on racial differences in medication adherence were conducted in the USA [51, 60, 72, 83, 86, 88–90, 104, 121, 123, 124, 127, 128, 131]. Two of these studies concluded that there were no racial differences in medication adherence [72, 123], while the remaining 13 studies found lower adherence for non-white ethnicities [51, 60, 83, 86, 88–90, 104, 121, 124, 127, 128, 131]. This association was found in black [51, 83, 86, 88–90, 128] and African-American [127], as well as in Latin-American and Hispanic subpopulations [88, 121, 131]. Similar results were found in New Zealand where 5 studies reported that non-European ethnicities resulted in lower medication adherence [35, 37, 122, 124, 126]. Other studies compared adherence differences between ethnic majorities and minorities in their countries (Table 3).

**Table 3:** Key findings

Key findings		
Study design		Number of study participants
✓ 73 cross-sectional studies		✓ 67 studies with fewer than 500 participants
✓ 23 cohort studies		✓ 18 studies with more than 500 participants but less than 5000
✓ 5 experimental studies		✓ 17 studies with more than 5000 participants
✓ and 1 case–control study		
**Continent**		**Type of treatment**
✓ 15 in Africa		✓ 72 studies investigated all types of medication used in T2DM^a^
✓ 42 in Asia		✓ 26 studies investigated oral antidiabetic medication
✓ 4 in South America,		✓ 4 studies investigated insulin
✓ 1 in Central America		
✓ 29 in North America		
✓ 5 in Europe		
✓ 6 in Oceania		

Type of socioeconomic factor	Economic and social factors	Ethnical and geographical factors
✓ Economic [61]	✓ Socioeconomic status	✓ Ethnicity/race
✓ Social [74]	✓ Occupational status	✓ Country of birth
✓ Ethnical/racial [19]	✓ Income	✓ Acculturation
✓ Geographical [18]	✓ Housing type	✓ Accessibility to healthcare
	✓ Insurance status	✓ Area of residence
	✓ Economic support	✓ Regional differences
	✓ Transportation availability	
	✓ Civil status	
	✓ Living arrangement	
	✓ Education	
	✓ Caste	
	✓ Religion	
	✓ Family support (social)	

Conclusions		
✓ The majority of studies found an association with antidiabetic medication adherence for two specific factors: insurance status [10] and ethnicity or race [18]		
✓ Other important factors were income and education		
✓ These factors may be taken into consideration when recommending treatments to patients and designing interventions to increase adherence		

^a^T2DM: Type 2 Diabetes Mellitus

#### Country of birth and acculturation

Two studies conducted in the USA investigated the association between country of birth and medication adherence [71, 85], 1 study determined that individuals born in the USA were less likely to be adherent [71], while the other found no difference in adherence between foreign- and local-born individuals with T2DM [85]. One Australian study found a positive association between a high degree of acculturation and adherence, and an inverse relation between beliefs in traditional Chinese medicine and medication adherence [39].

#### Accessibility to health care, area of residence and regional differences

A British study found that having higher indices of multiple deprivation (IMD) was associated with an increase in adherence [125], and these findings are in line with 1 US study on neighbourhood deprivation [77] and 1 New Zealand study [37]. Opposing these findings, another study conducted in New Zealand did not find any association between socioeconomic living area and medication adherence [126]. Three studies found that urban area of residence is associated with a decrease in adherence [45, 58, 104]; opposing these findings, 3 studies found that a greater distance to a healthcare provider is associated with a decrease in adherence [31, 61, 101]. Six studies found no difference in adherence by area of residence [31, 80, 98, 100, 122, 130]. Two studies from the USA found differences in adherence depending on the region of their home state [95, 104], while a Spanish and a Lebanese study found no association between the different regions in the country and medication adherence [41, 110].

## Discussion

### Findings in context

Previous reviews have concluded that many individuals with T2DM show poor adherence to their medication regimen [15–17].

A systematic review conducted by Azharuddin et al. (2021) found non-adherence to be more likely in employed individuals [20]. It should be noted that the review did not include studies where no association was found. In the current systematic review, 2 studies were found showing an association between employed individuals and decreased adherence [52, 53]. The majority [18] did not find any association between employment and medication adherence [25, 32, 36–51]. Other studies looking at antidiabetic medication adherence in unemployed [54–56], retired individuals [57] or housewives [33] led to inconclusive results, as the direction of associations were contradictory. These findings are in line with a systematic review conducted by Wibowo et al. (2022) that found no conclusive association between employment and level of adherence [17].

Azharuddin et al. (2022) also found that lower income was associated with less adherence [20]. In the current systematic review, 9 studies found a positive association between high income and higher adherence [42, 52, 59–65]. Fifteen studies [25, 37, 41, 44, 46, 51, 54, 58, 66–72] found no association between different levels of income and only 1 study [73] found an inverse association. The association trend between non-adherence and lower income was also supported by 6 studies [74–79] that found reduced adherence for individuals reporting financial hardship, as well as 2 studies [67, 81] concluding that higher medication cost was associated with lower adherence. In line with these findings, 4 studies [26–29] found individuals with higher socioeconomic status more likely to be adherent. To be noted is that for socioeconomic status 8 studies [28–35] found no association in either direction. Low income is also associated with low education, and it could be speculated that this may lead to poor disease knowledge and management.

Azharuddin et al. (2021) and Wibowo et al. (2022) found a positive association between higher education and better medication adherence in their reviews [17, 20]. In the current review, 40 of the studies investigating the association between education and antidiabetic medication adherence found no difference between the varying levels of education [25, 28, 30, 31, 37, 43–47, 49–52, 54, 56, 59–61, 67, 70–73, 75, 81, 82, 85, 97, 98, 101, 106–114]. A total of 17 studies [24, 26, 27, 36, 38, 41, 42, 53, 55, 63, 64, 91, 100, 102, 115–117] found a positive association between higher education and level of adherence, while only 1 study [74] reported a negative association. These findings were supported by studies where a tendency towards non-adherence in illiterate individuals was observed [48, 62, 100], as well as studies that found people with higher adherence more likely to have higher numeracy skills [72, 118] and better disease knowledge [62]. Lower education may be related to lower knowledge on the impact and severity of T2DM and the importance of adherence to medication regimens, suggesting that patients’ education should be considered as a relevant factor when trying to improve patients’ medication use.

Peeters et al. (2011) found no association between ethnicity/race and adherence in their systematic review [18]. Comparing their conclusions to the results found in the current systematic review, lower adherence may be associated with non-white ethnicity in the USA, which 13 [51, 60, 83, 86, 88–90, 104, 121, 124, 127, 128, 131] out of 15 studies [51, 60, 72, 83, 86, 88–90, 104, 121, 123, 124, 127, 128, 131] concluded. A similar association was found in New Zealand where all 5 studies conducted resulted in lower adherence for non-European ethnicities [35, 37, 122, 124, 126]. Ethnicity and race as a whole may be associated with lower income, lower socioeconomic status and lower education as well [132], which all are associated with lower adherence themselves. These results highlight the relevance of ethnicity when assessing individuals’ adherence to medication or more generally, to physicians’ recommendations. Hence, one could speculate that ethnicity does not only influence the biological mechanisms behind medication efficacy but also the way individuals access the healthcare system and manage their health conditions.

The current review showed that insurance status appeared to be associated with medication adherence in individuals with T2DM. This was shown by 10 studies including individuals with no [45, 65, 67, 76, 87, 89] or governmentally provided insurance [77, 86–88] which found associations with lower adherence. Supporting this tendency, 5 studies [65, 92–95] concluded that individuals with higher co-payments tend to have lower adherence and 1 study found that capitated health plans [90] are associated with lower adherence. Furthermore, 3 studies have shown that individuals with commercial insurance are more likely to have higher medication adherence [42, 58, 91]. One study [51] also concluded that having health coverage is associated with higher adherence. These findings are in line with a systematic review conducted in Asia [17]. Intuitively, insurance status is strongly related to socioeconomic aspects including educational level and income, particularly in countries where the access to care is not equally guaranteed to everyone. One could speculate that in such contexts disadvantaged socioeconomic groups are more prone to problematic medication adherence.

Area of residence may be associated with adherence to some degree as well, as 2 studies [45, 58] found lower adherence to be more likely in individuals living in urban environments, and 1 study [104] showed an association between living in a rural environment and higher adherence. Nonetheless, 6 studies [31, 80, 98, 100, 122, 130] did not find any association between antidiabetic medication adherence and area of residence. Additionally, distance to healthcare providers was investigated in 3 studies [31, 61, 101], all of which concluded that greater distances are associated with lower adherence.

Results related to other factors including housing type, economic support, transportation availability, caste, living arrangement, religion, family support (social), country of birth, acculturation and regional differences led to vague conclusions.

The literature investigating antidiabetic medication adherence and socioeconomic factors associated with it is quite extensive. A large heterogeneity of methodologies, study populations and designs were observed. Additionally, the same factor may be of different relevance depending on the social context in which the factor is viewed and considered. Furthermore, for socioeconomic factors such as income, education, ethnicity/race, as well as insurance status, it is important to highlight that they are associated with each other, and may also interact, particularly when the research interest lies in investigating life course epidemiology rather than cross-sectional associations. For example, a study conducted in the USA found that both income and race are independently associated with lower insurance coverage. It was also found that the combination of having low income and being part of a minority resulted in an considerably lower probability for being insured [133]. This socioeconomic construct should be thought of as a whole when practitioners consider whether an individual with T2DM would benefit from supportive measures for adherence improvement. Finally, multiple different self-perceived reasons for non-adherence were identified in other studies, those mainly being lack of faith in the effectiveness of their treatment [134], low perception of the consequences of diabetes [134, 135], forgetfulness [136], fear of injections [136] and embarrassment of public injections [137] (140).

The World Health Organization stated in its 2003 report on medication adherence that increasing the effectiveness of adherence may result in far greater impact on the health of the population than any improvement in specific medical treatments [11]. Despite the widespread prevalence of medication, non-adherence and its impact on patients’ well-being and life conditions, this aspect, which is ultimately related to medication efficacy, is often under-detected and undertreated. To enhance health and avoid complications in individuals with T2DM, it is of major public health relevance to detect non-adherence and improve treatment management. Generating evidence on associations between specific socioeconomic factors and antidiabetic medication non-adherence can help practitioners to target and intervene on individuals’ non-adherent behaviours, aiming to improve patients’ education with respect to T2DM course and management.

### Strengths

The large number of free-text search terms used for this systematic review increased inclusivity allowing to find a vast number of articles focusing on socioeconomic factors influencing antidiabetic medication adherence in individuals with T2DM. The thorough search enabled this systematic review to include the majority of articles on the topic in a global perspective. To the best of the authors knowledge, this is the first systematic review conducted under a global perspective on socioeconomic factors and antidiabetic medication adherence. Furthermore, a broader range of socioeconomic factors was investigated compared to other reviews, increasing knowledge on their associations with individuals’ adherence to antidiabetic medication.

### Limitations

The comparability of the studies found was limited by the non-uniformity of the factors investigated. Additionally, antidiabetic medication adherence was measured in different ways. Most studies found used a cross-sectional study design in which most often subjective measures of adherence were applied. The cross-sectional study design frequently comes with other sources of error as well, including small sample sizes, recall bias, as well as survey bias. Many cross-sectional studies found did not utilize a validated method for their questionnaire or interview, which may increase the bias further. Moreover, objective measures such as pill count or medication possession rate have their flaws, as they do not guarantee that the patient has actually taken the medication.

In terms of limitations concerning the methodology of the current systematic review, the quality assessment tool is open to some degree of subjective interpretation. Although two researchers conducted the quality assessment independently and discussed to reach a common agreement, the NHLBI’s tool does not provide a stringent rule for rating the studies.

### Conclusions and clinical implications

A range of socioeconomic factors associated with antidiabetic medication adherence was found. These factors may be taken into consideration when designing interventions to increase adherence. The studies included in this review vary greatly in terms of methodology, as well as quality. The factors such as insurance status and ethnicity/race consistently showed associations with antidiabetic medication adherence. Future research should aim to reduce the heterogeneity of the findings via a common validated methodology, allowing comparability between different studies.

Income, education, ethnicity/race and insurance are factors associated with adherence differences between patient groups, and these factors are also tightly intertwined with each other. When recommending treatments to patients, socioeconomic status should be considered, since increased awareness about differences and vulnerable groups may help to improve T2DM treatment management as a feasible short-term outcome, which in turn leads to enhancement of patients’ life quality in the long term.

### Supplementary Information

**Additional file 1: Appendix 1.** Exact searches of individual databases conducted on the 15.03.2022.

## Data Availability

Not applicable.
