# Supplementary material for: A global systematic overview of socioeconomic factors associated with antidiabetic medication adherence in individuals with type 2 diabetes
Source: J Health Popul Nutr. 2023 Nov 7;42:122. doi: 10.1186/s41043-023-00459-2 (PMC10631092; doi:10.1186/s41043-023-00459-2)
Supplement: Supplementary file 1 — Additional file 1: Appendix 1. Exact searches of individual databases conducted on the 15.03.2022. [file 41043_2023_459_MOESM1_ESM.docx]

# Supplementary Material to “A Global Systematic Overview of Socioeconomic Factors Associated with Antidiabetic Medication Adherence in Individuals with Type 2 Diabetes”

Christian Ming Studer^1^, Marie Linder^2^, Laura Pazzagli^3^

*^1^Department of Chemistry and Applied Biosciences, Institute for Pharmaceutical Sciences, ETH Zurich*

*^2^ Centre for Pharmacoepidemiology, Department of Medicine Solna, Karolinska Institutet*

*^3^ Clinical Epidemiology Division, Department of Medicine Solna, Karolinska Institutet*

# Appendix

## Appendix 1 - Exact searches of individual databases conducted on the 15.03.2022

| **PubMed** 2550 results  (Diabetes Mellitus, Type 2 [MeSH Terms] OR Hypoglycemic Agents [Pharmacological Action]OR Diabetes mellitus type 2[Title/Abstract] OR type 2 diabetes [Title/Abstract] OR diabetes type 2 [Title/Abstract] OR Diabetes mellitus type II[Title/Abstract] OR type II diabetes [Title/Abstract] OR diabetes type II [Title/Abstract] OR insulin-resistant diabetes[Title/Abstract] OR non-insulin dependent diabetes[Title/Abstract] OR adult-onset diabetes[Title/Abstract] OR metformin[Title/Abstract] OR biguanides[Title/Abstract] OR sulfonylureas[Title/Abstract] OR SGLT2 inhibitors[Title/Abstract] OR DPP-4 inhibitors[Title/Abstract] OR thiazolidinedione[Title/Abstract] OR GLP-1[Title/Abstract] OR insulin[Title/Abstract] OR alpha glucosidase inhibitors[Title/Abstract] OR blood glucose lowering drugs[Title/Abstract] OR antidiabetic medication[Title/Abstract])  AND (Socioeconomic Factors [MeSH Terms] OR socioeconomic factors[Title/Abstract] OR socioeconomic status[Title/Abstract] OR income[Title/Abstract] OR wealth[Title/Abstract] OR poverty[Title/Abstract] OR education[Title/Abstract] OR occupation[Title/Abstract] OR social class[Title/Abstract] OR marital status[Title/Abstract] OR civil status[Title/Abstract] OR ethnicity[Title/Abstract] OR country of origin[Title/Abstract] OR residential area[Title/Abstract] OR health literacy[Title/Abstract] OR health care accessibility [Title/Abstract])  AND (adherence [Title/Abstract] OR consistency [Title/Abstract] OR compliance [Title/Abstract] OR concordance [Title/Abstract] OR persistence [Title/Abstract] OR medication possession rate [Title/Abstract] OR drug use [Title/Abstract] OR discontinuation [Title/Abstract] OR interruption [Title/Abstract] OR medication use [Title/Abstract] OR Patient Compliance [MeSH Terms]) |
| --- |
| **Embase** 7,806 results  ('non insulin dependent diabetes mellitus'/exp OR 'antidiabetic agent'/exp OR 'diabetes mellitus type 2' OR 'type 2 diabetes' OR 'diabetes type 2' OR 'diabetes mellitus type ii' OR 'type ii diabetes' OR 'diabetes type ii' OR 'insulin-resistant diabetes' OR 'non-insulin dependent diabetes' OR 'adult-onset diabetes' OR 'metformin' OR 'biguanides' OR 'sulfonylureas' OR 'sglt2 inhibitors' OR 'dpp-4 inhibitors' OR 'thiazolidinedione' OR 'glp-1' OR 'insulin' OR 'alpha glucosidase inhibitors' OR 'blood glucose lowering drugs' OR 'antidiabetic medication')  AND ('socioeconomics'/exp OR 'socioeconomic status' OR 'social status'/exp OR 'religion'/exp OR 'education'/exp OR 'income' OR 'wealth' OR 'poverty' OR 'education' OR 'occupation' OR 'social class' OR 'marital status' OR 'civil status' OR 'ethnicity' OR 'country of origin' OR 'residential area' OR 'health literacy' OR 'health care accessibility')  AND ('patient compliance'/exp OR 'adherence' OR 'consistency' OR 'compliance' OR 'concordance' OR 'persistence' OR 'medication possession rate' OR 'drug use' OR 'discontinuation' OR 'interruption' OR 'medication use')  AND ([article]/lim OR [article in press]/lim OR [review]/lim) AND [humans]/lim AND [english]/lim AND [1990-2022]/py |
| **Web of Science** 4179 results  TS=((Diabetes mellitus type 2 OR Diabetes mellitus type 2 OR type 2 diabetes OR diabetes type 2 OR Diabetes mellitus type II OR type II diabetes OR diabetes type II OR insulin-resistant diabetes OR non-insulin dependent diabetes OR adult-onset diabetes OR metformin OR biguanides OR sulfonylureas OR SGLT2 inhibitors OR DPP-4 inhibitors OR thiazolidinedione OR GLP-1 OR insulin OR alpha glucosidase inhibitors OR blood glucose lowering drugs OR antidiabetic medication)  AND (socioeconomic factors OR socioeconomic status OR income OR wealth OR poverty OR education OR occupation OR social class OR marital status OR civil status OR ethnicity OR country of origin OR residential area OR health literacy OR health care accessibility)  AND (adherence OR consistency OR compliance OR concordance OR persistence OR medication possession rate OR drug use OR discontinuation OR interruption OR medication use)) |
| **WorldCat** 47 results  kw:(Diabetes mellitus type 2 OR Diabetes mellitus type 2 OR type 2 diabetes OR diabetes type 2 OR Diabetes mellitus type II OR type II diabetes OR diabetes type II OR insulin-resistant diabetes OR non-insulin dependent diabetes OR adult-onset diabetes OR metformin OR biguanides OR sulfonylureas OR SGLT2 inhibitors OR DPP-4 inhibitors OR thiazolidinedione OR GLP-1 OR insulin OR alpha glucosidase inhibitors OR blood glucose lowering drugs OR antidiabetic medication)  AND (socioeconomic factors OR socioeconomic status OR income OR wealth OR poverty OR education OR occupation OR social class OR marital status OR civil status OR ethnicity OR country of origin OR residential area OR health literacy OR health care accessibility)  AND (adherence OR consistency OR compliance OR concordance OR persistence OR medication possession rate OR drug use OR discontinuation OR interruption OR medication use) |
| **Bielefeld Academic Search Engine (BASE)** 546 results for articles and thesis with verbatim search  ("Diabetes mellitus type 2" OR "type 2 diabetes" OR "diabetes type 2" OR "Diabetes mellitus type II" OR "type II diabetes" OR "diabetes type II" OR "insulin-resistant diabetes" OR "non-insulin dependent diabetes" OR "adult-onset diabetes" OR "metformin" OR "biguanides" OR "sulfonylureas" OR "SGLT2 inhibitors" OR "DPP-4 inhibitors" OR "thiazolidinedione" OR "GLP-1" OR "insulin" OR "alpha glucosidase inhibitors" OR "blood glucose lowering drugs" OR "antidiabetic medication")  AND ("socioeconomic factors" OR "socioeconomic status" OR "income" OR "wealth" OR "poverty" OR "education" OR "occupation" OR "social class" OR "marital status" OR "civil status" OR "ethnicity" OR "country of origin" OR "residential area" OR "health literacy" OR "health care accessibility")  AND ("adherence" OR "consistency" OR "compliance" OR "concordance" OR "persistence" OR "medication possession rate" OR "drug use" OR "discontinuation" OR "interruption" OR "medication use") |
